# Morphophysiological and Anatomical Responses of Culantro (*Eryngium foetidum*) to In Vitro Salinity

**DOI:** 10.3390/plants15162522

**Published:** 2026-08-20

**Authors:** Haylson Rodrigues de Araújo, Juliane Maciel Henschel, Darlyara Reis Silva, Sérgio Heitor Sousa Felipe, Tiago Massi Ferraz, Fabrício de Oliveira Reis, Fábio Afonso Mazzei Moura de Assis Figueiredo, Thais Roseli Corrêa, Diego Silva Batista

**Affiliations:** 1 Programa de Pós-Graduação em Ciências Agrárias, Laboratório de Cultura de Tecidos, Universidade Estadual do Maranhão, Av. Lourenço Vieira da Silva, São Cristóvão, São Luís 65055-310, MA, Brazildarlyarareissilva.125@gmail.com (D.R.S.); sergio.h.s.felipe@gmail.com (S.H.S.F.); thaiscorrea@professor.uema.br (T.R.C.); 2 Universidade Federal Rural da Amazônia (UFRA), Campus Capitão Poço, Capitão Poço 68650-000, PA, Brazil; 3 Departamento de Botânica e Zoologia, Universidade Federal do Rio Grande do Norte, Natal 59078-970, RN, Brazil

**Keywords:** chlorophyll fluorescence, gas exchange, photosynthetic pigments, medicinal plant

## Abstract

Soil salinity is an increasing constraint to crop production, yet little is known about the responses of culantro (*Eryngium foetidum*), a medicinal and culinary species of high economic value, to saline conditions. This study evaluated the effects of NaCl-induced salinity (0, 40, and 80 mM) on the growth, photosynthetic performance, pigment content, and vascular anatomy of *E. foetidum* cultivated in vitro. After 45 days, salinity significantly reduced shoot and root length, leaf number, chlorophyll fluorescence, net CO_2_ assimilation, stomatal conductance, transpiration, carboxylation efficiency, and the contents of chlorophylls a and b, and carotenoids, with the strongest effects observed at 80 mM NaCl. In contrast, leaf area, biomass accumulation, specific leaf area, and intrinsic water-use efficiency were not significantly affected. Qualitative anatomical observations indicated apparent modifications in vascular organization under saline conditions, including narrower xylem vessels and phloem disorganization. Collectively, these findings demonstrate that salinity primarily impairs photosynthetic performance and vegetative growth while inducing morphological, physiological, and apparent anatomical responses in *E. foetidum* cultivated in vitro. This study provides the first integrated characterization of the responses of *E. foetidum* to in vitro salt stress, establishing a foundation for future investigations into the physiological mechanisms underlying salinity responses in this species.

## 1. Introduction

Soil salinity is a growing global concern because of its detrimental effects on plant growth and productivity. Abiotic stresses, including salinity, trigger a wide range of morphological, biochemical, and physiological responses in plants, affecting their growth, development, and productivity [1,2]. Elevated salinity levels can markedly reduce plant growth by inhibiting root development and intensifying oxidative stress. However, the magnitude of these effects varies considerably between species and genotypes, highlighting the need for species-specific strategies to mitigate salinity-induced damage [3].

Beyond growth inhibition, salinity also affects vegetative development by altering the expression of genes involved in the biosynthesis of secondary metabolites, including polyphenols and terpenoids, in plants exposed to saline conditions. These metabolic adjustments may influence both the nutritional quality of plant products and plants’ capacity to withstand environmental stress [4]. Conversely, recent studies by Li and Wang [5] have emphasized the remarkable phenotypic plasticity of plants in response to salinity, enabling adaptation through coordinated morphological modifications and biochemical adjustments. This adaptive response involves the reprogramming of metabolic pathways, particularly those regulating secondary metabolite biosynthesis, thereby optimizing resource allocation and minimizing the detrimental effects of salt stress [5].

Among the rich diversity of plant species native to Brazil, culantro (*Eryngium foetidum* L.) stands out for its unique phytochemical composition and broad range of applications. Its essential oil contains high concentrations of bioactive compounds, particularly (E)-2-dodecenal, dodecanoic acid, trans-2-dodecanoic acid, (E)-2-tridecenal, duraldehyde, and tetradecane [6,7]. In addition to its widespread culinary use [8], *E. foetidum* has long been employed in traditional medicine to treat influenza, pneumonia, diabetes, constipation, and other ailments [7]. The species is also recognized as a valuable source of vitamins and minerals [9]. Pharmacological studies have reported a wide range of biological activities, including anti-inflammatory, antioxidant, antimicrobial, anthelmintic, anticonvulsant, anticancer, antidiabetic, antimalarial, larvicidal, and hepatoprotective effects, underscoring its considerable phytochemical and economic potential.

In this context, plant biotechnology, particularly in vitro culture techniques, provides a powerful platform for investigating plant responses to salinity. According to Muchate et al. [10], in vitro cultivation enables direct assessment of salt stress under highly controlled conditions, minimizing environmental variability that often confounds field experiments. This approach facilitates a more precise understanding of the molecular and physiological mechanisms underlying salinity tolerance, including changes in gene expression and secondary metabolite biosynthesis [11,12].

Previous research supports the selection of *E. foetidum* for the present study by demonstrating its remarkable physiological and metabolic plasticity in response to agronomic practices, phenological stage, and abiotic stresses. Variations in fertilization strategies and leaf maturity have been shown to significantly alter the chemical profile of the species, particularly the concentration of the major constituent (E)-2-dodecenal [13]. Likewise, changes in secondary metabolite composition have been reported in plants subjected to water deficit and rehydration with carnitine [14], water deficit with or without rehydration, and well-watered plants treated with salicylic acid [15], as well as under drought stress with or without ascorbic acid supplementation [16].

Despite its recognized economic and pharmacological importance, little is known about *E. foetidum* responses to salinity, particularly under in vitro conditions. Therefore, this study aimed to evaluate the effects of salt stress on the morphology, anatomy, growth, and photosynthetic performance of *E. foetidum* cultivated in vitro. We hypothesized that salinity would induce coordinated morphological, anatomical, and physiological adjustments that modulate plant growth and photosynthetic performance, reflecting adaptive mechanisms that enable this species to cope with saline environments.

## 2. Results

**Figure 1:**
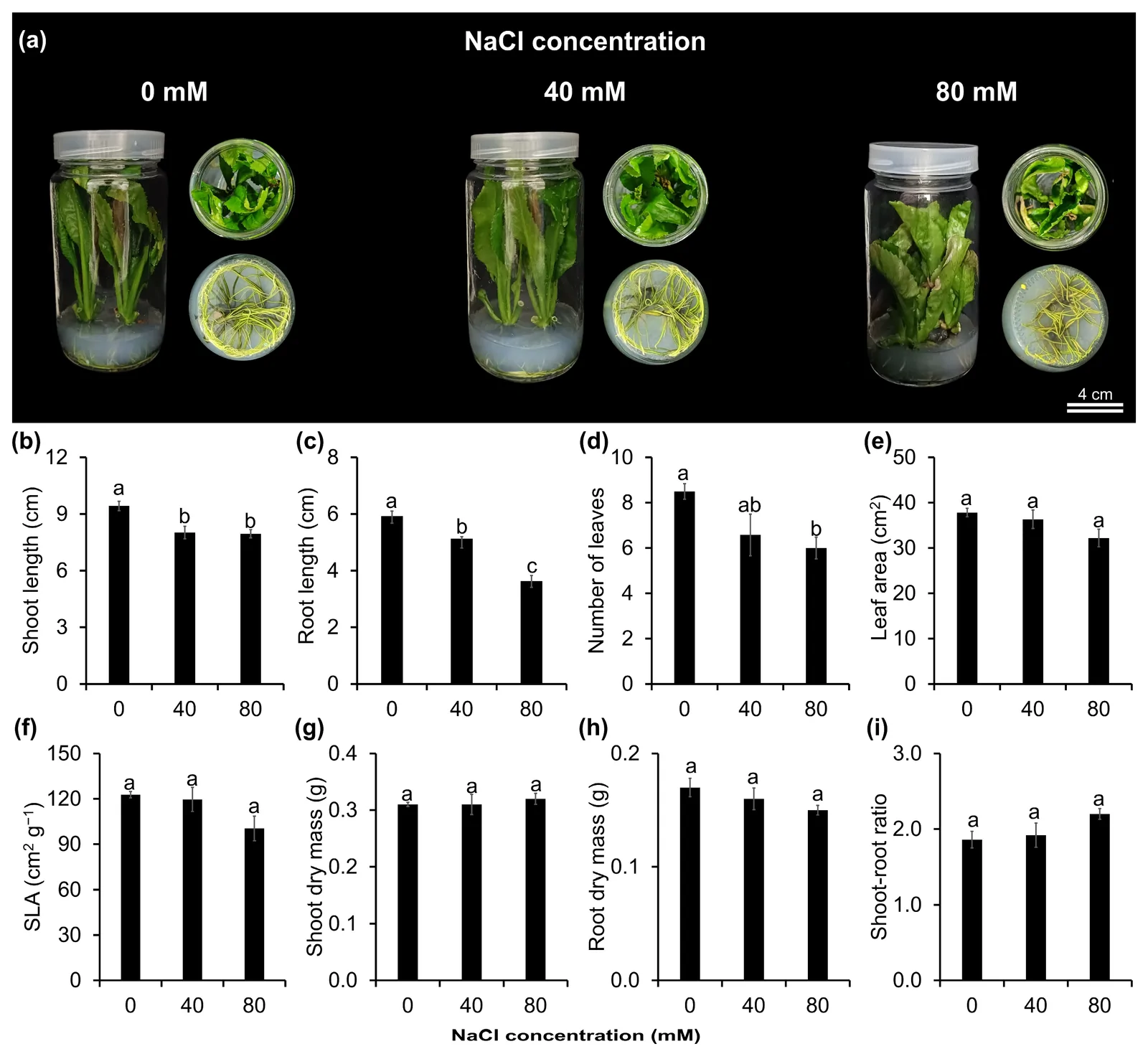
Phenotypic appearance and growth parameters of *Eryngium foetidum* plants after 45 days of in vitro cultivation under three salinity levels (0, 40, and 80 mM NaCl). (**a**) Pictures of representative plants; (**b**) shoot length (cm); (**c**) root length (cm); (**d**) number of leaves; (**e**) leaf area (cm^2^); (**f**) specific leaf area (cm^2^ g^−1^); (**g**) shoot dry mass (g); (**h**) root dry mass (g); and (**i**) shoot-to-root dry mass ratio. Values are presented as means ± standard error (*n* = 6). Different letters indicate significant differences among treatments according to Tukey’s test (*p* ≤ 0.05).

*Eryngium foetidum* plants exhibited symptoms of leaf senescence regardless of the in vitro salinity treatment (Figure 1). In contrast, plants exposed to NaCl showed a marked reduction in both shoot and root growth compared with the control, with the inhibitory effects becoming more pronounced as salinity increased and reaching their greatest intensity at 80 mM NaCl (Figure 1). Details of the analysis of variance are provided in Supplementary Table S1.

*E. foetidum* plants grown under different salinity levels showed no significant differences (*p* ≥ 0.05) in leaf area, specific leaf area, shoot dry mass, root dry mass, or shoot-to-root dry mass ratio (Figure 1e–i). In contrast, salinity significantly affected shoot length, decreasing it by 15% and 16% under 40 and 80 mM NaCl, respectively, compared with the control (Figure 1b). A similar response was observed for root length, which was reduced by 14% at 40 mM NaCl and by 39% at 80 mM NaCl (Figure 1c). Likewise, the number of leaves declined significantly, with reductions of 23% and 29% under 40 and 80 mM NaCl, respectively, relative to the control (Figure 1d).

**Figure 2:**
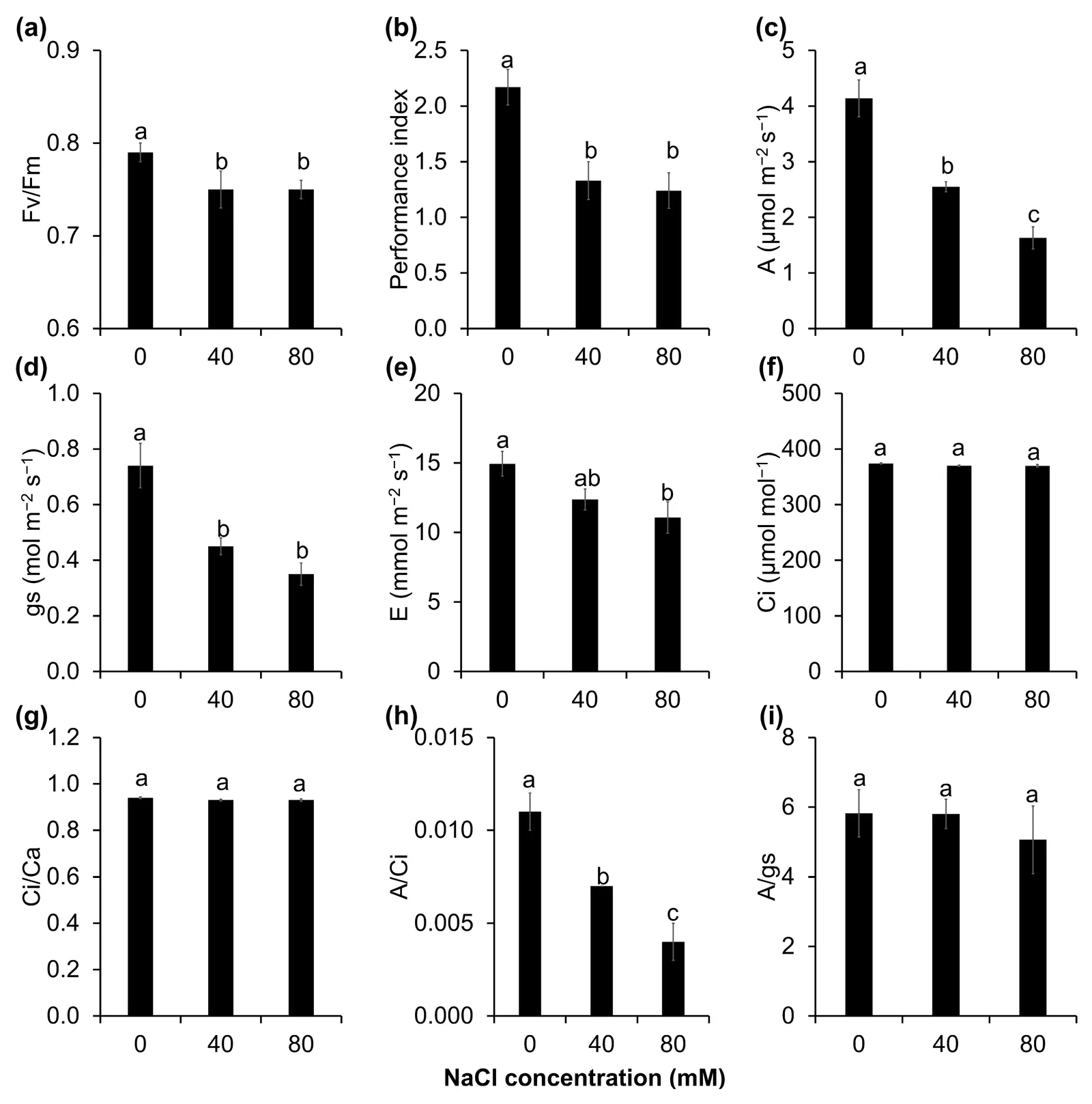
Chlorophyll a fluorescence and gas exchange parameters of *Eryngium foetidum* after 45 days of in vitro cultivation under different NaCl concentrations (0, 40, and 80 mM). (**a**) Maximum quantum yield of photosystem II (Fv/Fm); (**b**) performance index (PI); (**c**) net CO_2_ assimilation rate (A); (**d**) stomatal conductance (g_s_); (**e**) transpiration rate (E); (**f**) intercellular CO_2_ concentration (Ci); (**g**) ratio of intercellular to ambient CO_2_ concentration (Ci/Ca); (**h**) carboxylation efficiency (A/Ci); and (**i**) intrinsic water-use efficiency (A/g_s_). Values are presented as means ± standard error (*n* = 6). Different letters indicate significant differences among treatments according to Tukey’s test (*p* ≤ 0.05).

*E. foetidum* plants cultivated under 40 and 80 mM NaCl exhibited significant reductions in chlorophyll fluorescence parameters (Figure 2). The maximum quantum yield of photosystem II (Fv/Fm) decreased by approximately 6% under both salinity treatments compared with the control (Figure 2a). Similarly, the performance index (PI) declined markedly, with reductions of approximately 39% and 43% under 40 and 80 mM NaCl, respectively (Figure 2b).

Salinity also significantly impaired photosynthetic performance. Net CO_2_ assimilation rate (A) decreased by 38% and 61% in plants exposed to 40 and 80 mM NaCl, respectively (Figure 2c). Likewise, stomatal conductance (gs) was reduced by 40% and 53% under 40 and 80 mM NaCl, respectively (Figure 2d), followed by decreases of 17% and 26% in transpiration rate (E) (Figure 2e). Carboxylation efficiency (A/Ci) was also significantly affected, showing reductions of 38% and 60% in plants grown under 40 and 80 mM NaCl, respectively, compared with the control (Figure 2h). On the other hand, no significant differences (*p* > 0.05) were observed for intercellular CO_2_ concentration (Ci) (Figure 2f), ratio of intercellular to ambient CO_2_ concentration (Ci/Ca) (Figure 2g), and intrinsic water-use efficiency (A/gs) (Figure 2i).

**Figure 3:**
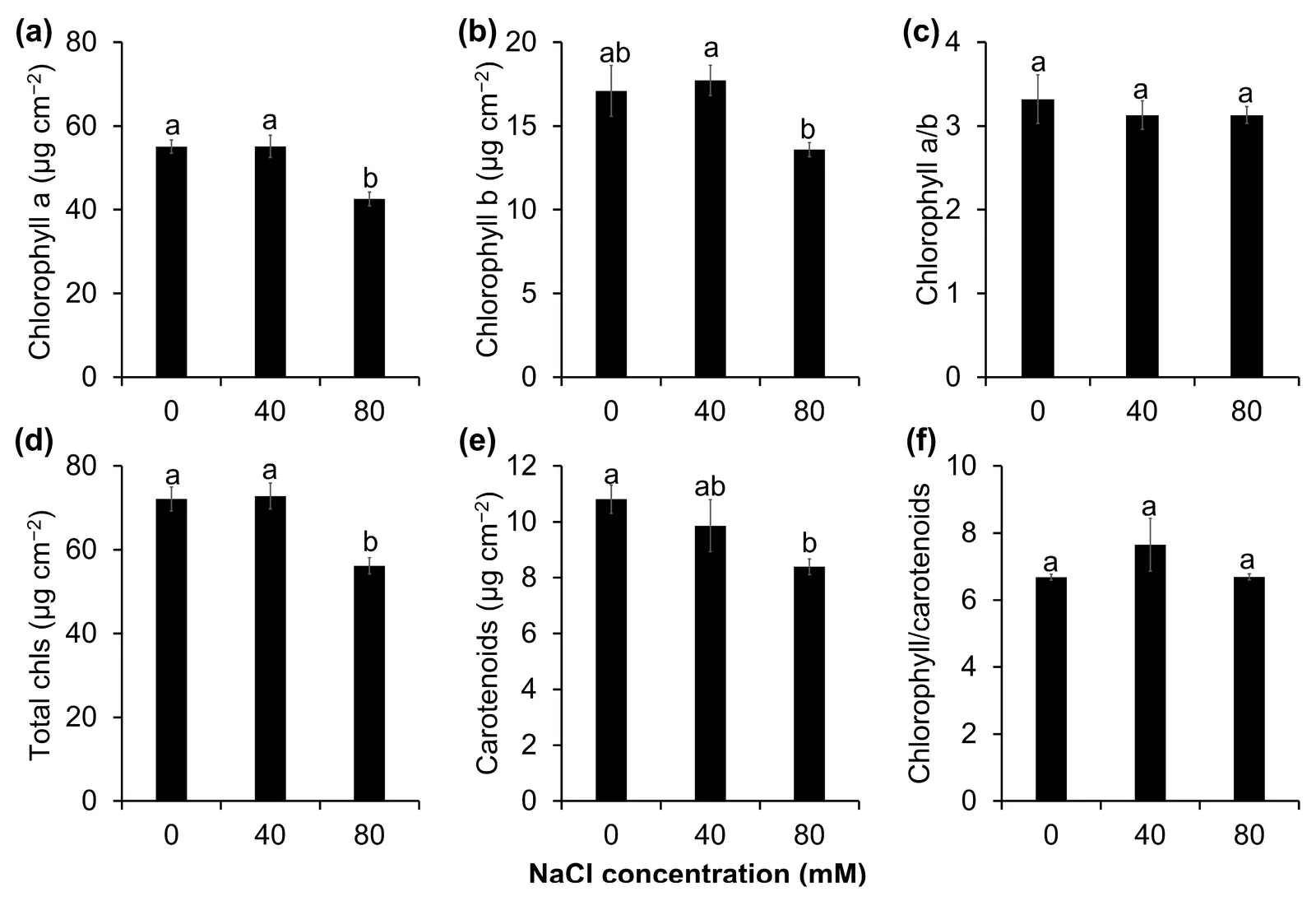
Photosynthetic pigment contents of *Eryngium foetidum* after 45 days of in vitro cultivation under different NaCl concentrations (0, 40, and 80 mM). (**a**) Chlorophyll a; (**b**) chlorophyll b; (**c**) chlorophyll a/b ratio; (**d**) total chlorophylls (chls); (**e**) carotenoids; and (**f**) total chlorophyll-to-carotenoid ratio. Values are presented as means ± standard error (*n* = 6). Different letters indicate significant differences among treatments according to Tukey’s test (*p* ≤ 0.05).

The concentration of photosynthetic pigments was decreased only under the highest salinity level (80 mM NaCl), significantly reducing the concentration of chlorophyll a by 23% (Figure 3a), chlorophyll b by 20% (Figure 3b), total chlorophylls by 22% (Figure 3d), and carotenoids by 22% (Figure 3e) compared with the control. In contrast, 40 mM NaCl did not affect these variables. Moreover, none of the NaCl treatments affected the chlorophyll a/b ratio (Figure 3c) and the total chlorophyll-to-carotenoid ratio (Figure 3f).

**Figure 4:**
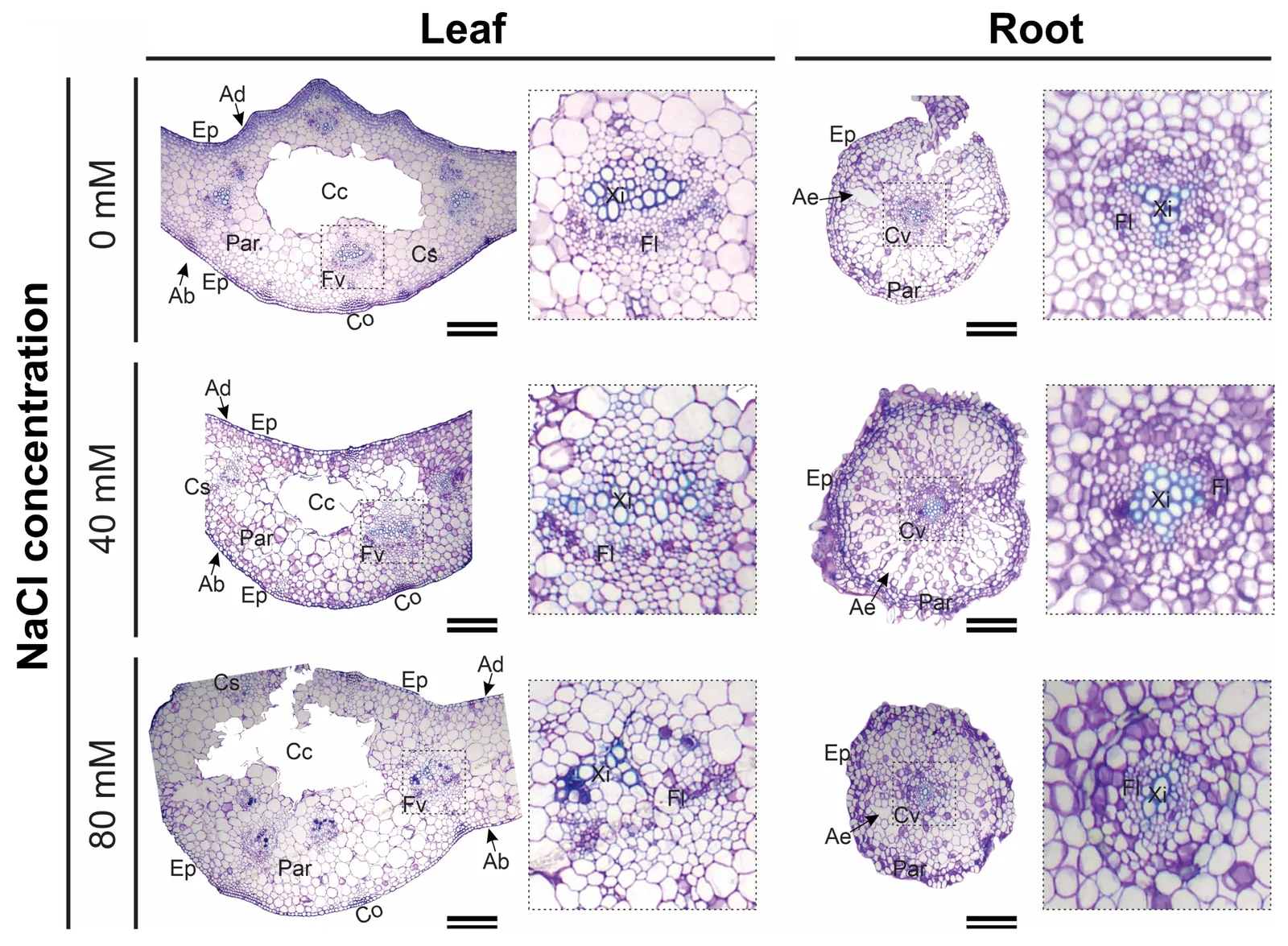
Representative transverse sections of the leaf midrib and root of *Eryngium foetidum* after 45 days of in vitro cultivation under different NaCl concentrations (0, 40, and 80 mM). Abbreviations: Ab, abaxial epidermis; Ad, adaxial epidermis; Ae, aerenchyma; Cc, central cavity; Co, collenchyma; Cs, secretory cavity; Cv, vascular cambium; Ep, epidermis; Fl, phloem; Fv, vascular bundle; Par, parenchyma; Xi, xylem. Scale bars = 300 μm.

Salt stress induced apparent structural changes in the vascular tissues of both leaves and roots (Figure 4). Control plants (0 mM NaCl) exhibited well-organized vascular bundles in both organs, with xylem vessels that appeared relatively wider based on qualitative observations. Plants exposed to 40 mM NaCl showed an apparent reduction in xylem vessel diameter in both leaves and roots, accompanied by visually more compact phloem tissue. These features were more evident under 80 mM NaCl, where xylem vessels appeared narrower, and the phloem exhibited apparent structural disorganization in both organs (Figure 4). Because these observations were based exclusively on qualitative microscopy, they should be interpreted as descriptive and require quantitative anatomical confirmation.

## 3. Discussion

Analysis of the morphological data indicates that NaCl exposure affected several growth traits of *E. foetidum*. Shoot and root length were significantly reduced under saline conditions, demonstrating that salinity negatively affected vegetative growth in this species. Although root length declined progressively with increasing NaCl concentration, shoot length did not differ significantly between the two saline treatments, indicating a trait-dependent rather than uniformly dose-dependent response. In contrast, leaf area, specific leaf area, shoot dry mass, root dry mass, and the shoot-to-root dry mass ratio were not significantly affected by salinity. However, leaf production was markedly reduced in salt-treated plants, with the greatest reduction observed at 80 mM NaCl, suggesting that salinity primarily restricted the formation of new organs rather than biomass accumulation.

Leaf senescence was observed in plants from all treatments, including the control, suggesting that this response was not exclusively associated with salt stress. Senescence under all culture conditions indicates that factors inherent to the in vitro environment may also have contributed to this response. Previous studies have shown that limited gas exchange within sealed culture vessels can alter the internal gaseous environment, including ethylene accumulation, which has been associated with premature leaf senescence and other physiological disorders in tissue-cultured plants [17]. Although the present study did not evaluate ethylene concentration or the internal gaseous environment, these factors may have contributed to the observed senescence and should be investigated in future studies.

Collectively, these findings indicate that salinity substantially altered the morphology of *E. foetidum* cultivated in vitro, primarily by restricting shoot and root elongation and reducing leaf production, while having little effect on biomass accumulation. This pattern suggests that the species exhibits phenotypic plasticity, reallocating growth under saline conditions without markedly compromising final dry biomass during the experimental period. Similar responses have been reported in other plant species exposed to salinity, where stress inhibits shoot, root, and leaf development, alters root system architecture by producing shorter and less-branched roots with reduced capacity for water and nutrient uptake, and frequently decreases fresh and dry biomass as a consequence of impaired plant growth [17,18,19,20,21,22].

Plant growth under saline conditions is generally characterized by two distinct phases: an initial osmotic phase, which restricts the expansion of young leaves, followed by a second phase associated with ionic toxicity that accelerates leaf senescence [23]. During the early stages of salt stress, the osmotic component predominates, reducing plant growth similarly to water deficit, likely through root-derived hormonal signaling that regulates shoot development [24].

The reductions in shoot and root length observed in the present study are consistent with the osmotic effects of salinity, which lower the external water potential and limit water uptake by the root system [25]. In addition, salt stress inhibits cell division and cell expansion, thereby restricting the elongation of vegetative organs [25]. The reduction in leaf number may also result from the progressive accumulation of NaCl in older leaves, where excessive ion accumulation in the cell wall and cytoplasm limits tissue growth and accelerates senescence, representing an adaptive strategy to preserve younger tissues under saline conditions [26].

To mitigate salt-induced damage, plants activate a range of protective mechanisms, including accumulating compatible solutes such as proline, glycine betaine, and polyols, which contribute to osmotic adjustment while stabilizing proteins and cellular membranes against dehydration [25]. Salt tolerance is further enhanced through activation of antioxidant systems, accumulation of polyamines and nitric oxide, and modulation of phytohormone signaling pathways [27]. These protective responses may explain the maintenance of leaf area and dry biomass observed in the present study despite reductions in organ growth, suggesting that *E. foetidum* partially compensated for the adverse effects of salinity during the experimental period. Supporting this interpretation, Manjunatha et al. [28] reported a significant increase in L-proline accumulation in *E. foetidum* exposed to nickel stress, indicating that osmotic adjustment may also contribute to the tolerance of this species to salinity. Nevertheless, further studies are needed to identify the specific osmolytes and defense mechanisms involved in its salt stress response.

In addition to osmotic stress, salinity promotes excessive production of reactive oxygen species (ROS), leading to oxidative damage that negatively affects plant growth and metabolism [29]. However, stress-induced signaling pathways, particularly those mediated by abscisic acid (ABA), play a central role in coordinating adaptive responses, allowing key physiological processes to be maintained under adverse environmental conditions [30].

Analysis of photosynthetic pigments revealed significant reductions only under the highest salinity level (80 mM NaCl), indicating that severe salt stress directly impairs pigment biosynthesis. Chlorophyll a, chlorophyll b, total chlorophyll, and carotenoid contents were all significantly reduced, whereas neither the chlorophyll a/b ratio nor the total chlorophyll-to-carotenoid ratio was affected. The stability of these pigment ratios despite the decline in their absolute concentrations suggests that salinity caused a proportional reduction in pigment accumulation rather than altering the relative composition of the photosynthetic apparatus.

Chlorophylls and carotenoids are essential components of the photosynthetic machinery, responsible for harvesting light energy and protecting the photosystems from photooxidative damage [31]. Consequently, the reduction in pigment content observed under severe salinity is likely to have contributed to the impairment of photosynthetic performance in *E. foetidum*. Similar responses have been reported by Hawrylak-Nowak et al. [32], who demonstrated that declines in photosynthetic pigments not only reduce photosynthetic efficiency but also intensify oxidative stress, ultimately disrupting physiological homeostasis and limiting plant growth and development. These findings align with the reductions in chlorophyll fluorescence and gas exchange observed in the present study, reinforcing the conclusion that photosynthetic metabolism is a primary target of salt stress in *E. foetidum*.

Hamani et al. [33] likewise identified salinity as a major factor limiting photosynthetic pigment accumulation, reporting significant reductions in chlorophyll content in in vitro-grown cotton plants exposed to increasing NaCl concentrations. Azzam et al. [21] reported similar findings in *Stevia rebaudiana* (Bertoni), confirming that saline conditions impair pigment biosynthesis across different species cultivated in vitro. These observations are consistent with the present study, where only the highest salinity level (80 mM NaCl) significantly reduced chlorophyll and carotenoid contents, indicating that severe salt stress is required to disrupt pigment metabolism in *E. foetidum*.

The chlorophyll a fluorescence results further demonstrate that salinity adversely affected the photosynthetic apparatus. Both the maximum quantum yield of photosystem II (Fv/Fm) and the performance index (PI) declined significantly in plants exposed to NaCl, indicating progressive impairment of PSII photochemistry. Because chlorophyll fluorescence provides a sensitive, non-destructive indicator of photosynthetic performance, these responses reveal that salt stress compromises the efficiency of the photosynthetic machinery before severe external symptoms become evident. Understanding these physiological adjustments is therefore essential for elucidating the mechanisms of salt tolerance and for developing strategies to improve plant performance under saline conditions [34,35].

Previous studies on *E. foetidum* support this species’ sensitivity to salinity. Ewase et al. [36] reported that increasing NaCl concentrations reduced seed germination and inhibited all major growth parameters. Likewise, Zidan [37] showed that exposure to 80 and 120 mM NaCl induced marked accumulation of proline and other free amino acids, suggesting that osmotic adjustment is an important adaptive response in this species. Together, these findings reinforce the view that *E. foetidum* responds to salt stress through coordinated physiological and biochemical adjustments, although these mechanisms appear insufficient to fully prevent growth inhibition under severe salinity.

The combined reductions in photosynthetic pigments and chlorophyll fluorescence observed in the present study provide strong evidence that salinity impaired the photosynthetic apparatus of *E. foetidum*. Nevertheless, these changes were not accompanied by significant reductions in final biomass, suggesting that adaptive mechanisms partially compensated for the physiological damage during the experimental period. The decline in total chlorophyll was mainly driven by reductions in chlorophyll a, which is generally considered more susceptible to salt stress than chlorophyll b [38]. According to Zhang et al. [39], this reduction is associated with structural damage to chloroplasts, including swelling and disruption of thylakoid membranes and the chloroplast envelope caused by excessive ion accumulation, which also interferes with chlorophyll biosynthesis. In addition, salt stress induces a broad range of physiological and biochemical responses, including ion exclusion and compartmentalization, osmolyte accumulation, alterations in photosynthetic pathways and membrane organization, activation of antioxidant enzymes, and hormonal regulation [40]. Collectively, these responses influence PSII functionality and overall photosynthetic efficiency. To minimize cellular damage, plants also activate antioxidant defense systems and hormonal signaling pathways [24], while restricting Na^+^ and Cl^−^ transport to leaves and sequestering these ions into vacuoles. Such compartmentalization prevents excessive cytoplasmic ion accumulation and consequently reduces salt toxicity [41].

Gas exchange measurements further demonstrated that salinity significantly reduced net CO_2_ assimilation (A), stomatal conductance (gs), transpiration (E), and carboxylation efficiency (A/Ci). The simultaneous decreases in A, gs, and E indicate that stomatal closure was an important component of the salt-stress response. However, the absence of significant changes in intercellular CO_2_ concentration (Ci) suggests that reduced stomatal aperture did not substantially limit CO_2_ availability within the leaf. Instead, the pronounced decline in carboxylation efficiency points to important non-stomatal limitations, including reduced biochemical capacity for CO_2_ fixation. These physiological impairments are consistent with the reductions in plant growth observed in Figure 2 and may be associated with the accumulation of Na^+^ and Cl^−^, which are known to promote stomatal closure and disrupt photosynthetic metabolism [42]. Furthermore, plants exposed to 80 mM NaCl exhibited a marked reduction in transpiration, reflecting partial stomatal closure. This response may also have been reinforced by the greater leaf thickness observed under severe salinity, as thicker leaves generally exhibit lower transpiration rates because of increased resistance to water loss.

In general, the first physiological response of plants to salinity is osmotic stress, which rapidly triggers stomatal closure. Although this response reduces water loss, it also restricts photosynthetic activity by limiting CO_2_ assimilation [43]. Gas exchange impairment under saline conditions is therefore largely associated with stomatal dysfunction, as ionic imbalance (particularly competition between Na^+^ and K^+^) disrupts guard cell regulation, reducing CO_2_ uptake and transpirational efficiency [44]. Moreover, excessive salt accumulation in leaf tissues damages both guard cells and mesophyll cells, further restricting gas diffusion and contributing to the decline in photosynthetic performance [23,44]. These mechanisms collectively explain the coordinated reductions in chlorophyll fluorescence, photosynthetic pigment content, and gas exchange observed in *E. foetidum* under salt stress.

According to Boussora et al. [45], plants exposed to salt stress regulate stomatal aperture to balance CO_2_ uptake and water loss, thereby maintaining photosynthetic activity while minimizing excessive dehydration. These authors also reported that salinity often increases intrinsic water-use efficiency because transpiration decreases proportionally more than carbon assimilation. In the present study, however, intrinsic water-use efficiency remained unchanged across treatments, despite significant reductions in stomatal conductance and transpiration. This stability suggests that *E. foetidum* maintained a relatively balanced relationship between carbon gain and water loss, reflecting an important physiological adjustment that contributes to homeostasis under saline conditions.

Salt stress also induced apparent anatomical modifications in the vascular tissues of both roots and leaves. Qualitative observations suggested a progressive reduction in the apparent diameter of xylem vessels with increasing salinity. Although these observations were based solely on visual comparisons and were not supported by quantitative anatomical measurements, they are consistent with previous reports describing reduced xylem vessel dimensions in plants exposed to osmotic stress. Narrower xylem vessels have been associated with a lower susceptibility to embolism and improved hydraulic safety under water-limited conditions. However, because the present study did not include quantitative anatomical analyses, these observations should be regarded as preliminary and require confirmation through detailed morphometric analyses in future investigations.

Comparable anatomical responses have been reported in other species exposed to salinity. In wheat (*Triticum aestivum* L.) irrigated with diluted seawater, Nassar et al. [46] observed reductions in vascular bundle area and xylem vessel diameter, changes that were associated with impaired ion transport. Ionic imbalance resulting from excessive salt accumulation can disrupt cellular metabolism, photosynthesis, and root architecture. Nevertheless, plants have evolved mechanisms to maintain ion homeostasis through selective ion transport and intracellular compartmentalization, thereby reducing the detrimental effects of salt stress [47].

Another noteworthy observation was the apparent increase in cell wall lignification under saline conditions. Although lignin content was not quantified, anatomical sections of salt-treated plants showed darker, thicker cell walls than those of the control, suggesting enhanced lignification. Lignin deposition is a common response to environmental stress and contributes to increased cell wall rigidity and tissue thickening [48]. Enhanced lignification of vascular tissues may improve their mechanical resistance, particularly when plants reduce root water potential to sustain water uptake under osmotic stress [49]. Similar anatomical adjustments have been described in *Lycium barbarum* L., where increasing salinity promoted thickening of xylem and phloem tissues, potentially enhancing water and nutrient transport while reducing water loss [17]. Likewise, changes in xylem vessel thickness associated with lignin deposition have been reported in rice (*Oryza sativa*), supporting the hypothesis that vascular reinforcement represents an important structural adaptation to saline environments [49].

## 4. Materials and Methods

### 4.1. Plant Material and In Vitro Establishment

*Eryngium foetidum* plants were established through the in vitro germination of seeds collected from a single mother plant originating from São Luís, Maranhão, Brazil. Thus, the experimental material consisted of different seed-derived individuals belonging to the same maternal family rather than a single clonal genotype. The experiment was conducted at the Plant Tissue Culture Laboratory of the State University of Maranhão (2°34′57″ S, 44°12′56″ W).

Seeds were surface-sterilized by immersion in 70% (*v*/*v*) ethanol for 1 min, followed by immersion in 2% (*w*/*v*) sodium hypochlorite (NaClO) for 15 min, then rinsed three times with sterile distilled water to remove residual disinfectants. The disinfected seeds were inoculated into glass test tubes (25 × 150 mm) containing semisolid Murashige and Skoog (MS) basal medium supplemented with MS salts and vitamins [50]. The medium was supplemented with 30 g L^−1^ sucrose (Dinâmica^®^, Jardim da Glória, SP, Brazil) and solidified with 2.0 g L^−1^ of Phytagel (Sigma-Aldrich^®^, St. Louis, MO, USA). No plant growth regulators were added to the culture medium. The pH was adjusted to 5.7 ± 0.1 before autoclaving at 121 °C and 1.1 atm for 20 min. After seed germination and seedling establishment, nodal segments (~2 cm long) were excised and transferred to 350-mL glass culture vessels containing 60 mL of the same semisolid MS medium supplemented with three NaCl concentrations (0, 40, or 80 mM). Because no previous studies have investigated salinity responses in *E. foetidum* under in vitro conditions, the NaCl concentrations were selected based on preliminary experiments conducted in our laboratory to establish moderate and severe, yet non-lethal, levels of salt stress. Sodium chloride was added to the culture medium before pH adjustment and autoclaving. Two nodal segments were cultured per vessel, which was considered the experimental unit. The vessels were closed with rigid polypropylene caps, with two holes (10 mm) covered by microporous tape membranes, as proposed by [51], and maintained in a growth room at 25 ± 2 °C under a 16-h photoperiod with a photosynthetic photon flux density of 96 μmol m^−2^ s^−1^. Plant growth, anatomical, and physiological evaluations were performed after 45 days of culture.

### 4.2. NaCl Treatments and Experimental Design

Nodal segments (approximately 2 cm in length) were cultured in 350 mL glass jars containing 60 mL of semisolid MS basal medium prepared under the same conditions described for in vitro establishment. Three salinity levels were evaluated by supplementing the culture medium with NaCl at concentrations of 0, 40, and 80 mM. Two explants were inoculated into each culture vessel. The cultures were maintained in a growth chamber at 25 ± 2 °C under a 16 h photoperiod with a photosynthetic photon flux density of 96 μmol m^−2^ s^−1^. Growth, physiological, and chemical analyses were performed after 45 days of in vitro cultivation. The experiment was conducted in a completely randomized design with three NaCl concentrations (0, 40, and 80 mM) and 12 experimental units per treatment. Each experimental unit consisted of one culture vessel containing two explants. The culture vessel was considered the biological replicate because both explants shared the same culture medium and headspace. For the analysis presented in Figure 1, Figure 2 and Figure 3, six experimental units were randomly selected from each treatment (*n* = 6). When measurements were obtained from both explants within a vessel, the values were averaged to generate a single observation for statistical analysis.

### 4.3. Plant Growth Parameters

The following growth parameters were evaluated: plant height (cm), longest root length (cm), number of leaves, leaf area (cm^2^), specific leaf area (cm^2^ g^−1^)—obtained by dividing total leaf area per plant by leaf dry mass, shoot dry mass (g), root dry mass (g), and the shoot-to-root dry mass ratio. Plant height and longest root length were measured using a graduated ruler. For dry mass determination, shoots and roots were separated, individually placed in Kraft paper bags, and dried in a forced-air oven at 45 °C until constant weight was reached, using a precision balance. The shoot-to-root dry mass ratio was subsequently calculated from the dry biomass data. Leaf area was determined using the ImageJ^®^ software, version 1.43 [52].

### 4.4. Chlorophyll a Fluorescence

Chlorophyll a fluorescence measurements were performed on the third fully expanded leaf from the apex of each plant. The maximum quantum yield of photosystem II (Fv/Fm) and the performance index (PI) were evaluated. Measurements were obtained using a portable non-modulated fluorometer (Pocket PEA, Hansatech Instruments, Norfolk, UK). Before analysis, leaves were dark-adapted for 30 min using specific leaf clips to ensure complete opening of the reaction centers, minimal heat dissipation, and full oxidation of the primary quinone electron acceptor [53].

### 4.5. Photosynthetic Pigments

Photosynthetic pigment contents were determined spectrophotometrically following extraction with dimethyl sulfoxide (DMSO). Five leaf discs (5 mm diameter) were collected from the third fully expanded leaf of each plant and placed in test tubes containing 5 mL of DMSO. Samples were incubated in the dark at 25 °C for 48 h. Absorbance was measured at 480, 645, and 665 nm using a UV–Vis spectrophotometer (BEL Engineering, Monza, Italy; UV-M51 UV/Vis Spectrophotometer). Chlorophyll a, chlorophyll b, and carotenoid contents were calculated according to the equations proposed by Wellburn [54]:

- Chlorophyll a (μg mL^−1^) = (12.19 × A665) − (3.45 × A649)
- Chlorophyll b (μg mL^−1^) = (21.99 × A649) − (5.32 × A665)
- Carotenoids (μg mL^−1^) = [(1000 × A480) − (2.14 × chlorophyll a) − (70.16 × chlorophyll b)]/220

### 4.6. Gas Exchange

Gas-exchange measurements were performed between 08:00 a.m. and 10:00 a.m. (approximately 2 h after the beginning of the photoperiod) using a portable infrared gas analyzer (LI-6400XT, LI-COR Biosciences, Lincoln, NE, USA). Plants were removed from the culture vessels immediately before the measurements, and fully expanded, healthy leaves were selected for analysis. The evaluated parameters included net CO_2_ assimilation rate (A), stomatal conductance (g_s_), transpiration rate (E), the ratio of intercellular to ambient CO_2_ concentration (Ci/Ca), intrinsic water-use efficiency (A/g_s_), and instantaneous carboxylation efficiency (A/Ci). Measurements were performed at a reference CO_2_ concentration of 400 µmol mol^−1^, an air flow rate of 500 µmol s^−1^, relative humidity of 60 ± 5%, and leaf temperatures ranging from 28 to 31 °C. The photosynthetic photon flux density inside the chamber was set to 1000 µmol m^−2^ s^−1^ using the LI-6400XT red-blue LED light source (10% blue light). This irradiance was selected to provide light-saturating conditions during gas-exchange measurements and has been successfully employed in previous studies with in vitro-grown plants using the same measurement protocol, without evidence of photoinhibitory effects [55]. Moreover, preliminary assessments conducted in our laboratory have consistently indicated that this PPFD does not induce photoinhibition under our experimental conditions.

### 4.7. Anatomical Analysis

To characterize the anatomical responses of *E. foetidum* plants cultivated in vitro for 45 days under different salinity levels, samples of the midrib from the third fully expanded leaf and root segments from the median region were collected and fixed in FAA solution (formaldehyde:acetic acid:50% ethanol; 1:1:18, *v*/*v*/*v*) for 48 h [56]. After fixation, the samples were dehydrated through a graded ethanol series (10, 20, 30, 40, 50, 60, 70, 80, 85, 90, 95, and 100%). Samples were maintained for 45 min in ethanol concentrations ranging from 30 to 70% and for 2 h in concentrations from 80 to 100%. The dehydrated material was subsequently subjected to pre-infiltration and infiltration procedures before being embedded in acrylic resin (Historesin^®^, Leica Instruments, Jena, Germany). The embedded samples were sectioned transversely at 7 μm using a semi-automatic rotary microtome (MRP2015, Lupetec Tecnologia Aplicada, São Carlos, SP, Brazil). The sections were stained with toluidine blue (pH 3.2) for 8 min [57], mounted on permanent slides, and examined under a light microscope (B20T, Bioptika, Colombo, PR, Brazil) equipped with a U-photo imaging system, a digital camera (CMOS-5.0, Bioptika, Colombo, PR, Brazil), and Capture V2.1 software for image acquisition.

### 4.8. Data Analysis

The experiment used a completely randomized design with three NaCl concentrations (0, 40, and 80 mM). The culture vessel, containing two explants, was considered the experimental unit. Quantitative data were initially assessed for normality using the Shapiro–Wilk test and homogeneity of variances using Levene’s test. Data satisfying these assumptions were subjected to one-way analysis of variance (ANOVA), and treatment means were compared using Tukey’s test at the 5% probability level (*p* ≤ 0.05). All statistical analyses were performed using the Genes software package, version 1990.2026.3 [58].

## 5. Conclusions

Salinity negatively affected the morphophysiology of *Eryngium foetidum* cultivated in vitro, causing significant reductions in shoot and root growth, leaf production, photosynthetic performance, chlorophyll fluorescence, gas exchange, and photosynthetic pigment contents. In contrast, leaf area, biomass accumulation, and specific leaf area were not significantly affected during the experimental period. Salt stress also induced apparent qualitative modifications in the vascular tissues, including narrower xylem vessels and phloem disorganization, although these observations require quantitative anatomical confirmation. Collectively, these findings provide the first integrated characterization of the morphological, physiological, and anatomical responses of *E. foetidum* to salinity under in vitro conditions, providing a valuable basis for future studies aimed at elucidating the mechanisms underlying salinity responses and supporting the development of cultivation strategies for this economically and medicinally important species.

## Data Availability

The original contributions presented in the study are included in the article, further inquiries can be directed to the corresponding authors.

## Supplementary Materials

The following supporting information can be downloaded at: https://www.mdpi.com/article/10.3390/plants15162522/s1, Table S1: Summary of the analysis of variance of *Eryngium foetidum* plants after 45 days of in vitro cultiva-tion under three salinity levels (0, 40, and 80 mM NaCl).
