# Supplementary material for: Morphophysiological and Anatomical Responses of Culantro (Eryngium foetidum) to In Vitro Salinity
Source: Plants (Basel). 2026 Aug 20;15(16):2522. doi: 10.3390/plants15162522 (PMC13516618; doi:10.3390/plants15162522)
Supplement: Supplementary file 1 [file plants-15-02522-s001.zip › plants-4490431-supplementary.pdf]

Supplementary Information

Morphophysiological and anatomical responses of culantro (*Eryngium foetidum*) to in vitro salinity

Haylson Rodrigues de Araújo[1], Juliane Maciel Henschel[1,*], Darlyara Reis Silva[1], Sérgio Heitor Sousa Felipe[1,2], Tiago Massi Ferraz[1], Fabrício de Oliveira Reis[1], Fábio Afonso Mazzei Moura de Assis Figueiredo[1], Thais Roseli Corrêa[1], Diego Silva Batista[1,3,*]

1 Programa de Pós-graduação em Ciências Agrárias, Laboratório de Cultura de Tecidos, Universidade Estadual do Maranhão, Av. Lourenço Vieira da Silva - São Cristóvão, São Luís, Maranhão - Brazil, 65055-310; eng.haylson@hotmail.com (H.R.A); darlyarareissilva.125@gmail.com (D.R.S.); sergio.h.s.felipe@gmail.com (S.H.S.F.); ferraztm@gmail.com (T.M.F.); fareoli@gmail.com (F.d.O.R.); figueiredo.uema@gmail.com (F.A.M.M.d.A.F.); thaiscorrea@professor.uema.br (T.R.C.);

2 Universidade Federal Rural da Amazônia (UFRA), Campus Capitão Poço, Capitão Poço, PA, 68650-000, Brazil;

3 Departamento de Botânica e Zoologia, Universidade Federal do Rio Grande do Norte, Natal, RN 59078-970, Brazil;

* Correspondence: julianemhenschel@gmail.com (J.M.H.) and diego.batista@ufrn.br (D.S.B.)

Supplementary Table S1. Summary of the analysis of variance of *Eryngium foetidum* plants after 45 days of in vitro cultivation under three salinity levels (0, 40, and 80 mM NaCl). DF: degrees of freedom; RMS: residual mean square; Fcal: F calculated using the F test. ^{ns}: non-significant at a 5% probability; * and ** significant at a level of 5 and 1% probability, respectively, by the F test.

**Variables**	**DF**	**RMS**	**Fcalc**	**Probability (%)**
Shoot length	2	4.211	9.512	0.21**
Root length	2	8.170	59.58	0.00**
Number of leaves	2	10.264	4.083	3.84*
Leaf area	2	50.889	2.711	9.87^{ns}
Specific leaf area	2	877.758	3.129	7.31^{ns}
Shoot dry mass	2	0.001	0.639	100.00^{ns}
Root dry mass	2	0.001	2.102	15.67^{ns}
Shoot/root ratio	2	0.203	2.558	11.06^{ns}
Fv/Fm	2	0.004	5.279	1.83*
PI	2	1.561	9.746	0.19**
A	2	8.054	10.958	0.001**
g_s	2	0.207	13.56	0.083
E	2	19.401	4.406	3.67^{ns}
Ci	2	28.538	2.0375	17.30^{ns}
Ci/Ca	2	0.001	2.272	14.56^{ns}
A/Ci	2	0.001	29.341	0.001**
A/g_s	2	0.939	0.358	100.00^{ns}
Chlorophyll *a*	2	261.12	12.94	0.10**
Chlorophyll *b*	2	24.68	4.50	3.48*
Chlorophyll *a/b*	2	0.055	0.274	100.00^{ns}
Total chlorophylls	2	444.87	12.50	0.11**
Carotenoids	2	7.436	3.737	5.47^{ns}
Chlorophyll/carotenoids	2	1.553	1.435	26.99^{ns}
